# Association between warfarin and COVID-19-related outcomes compared with direct oral anticoagulants: population-based cohort study

**DOI:** 10.1186/s13045-021-01185-0

**Published:** 2021-10-19

**Authors:** The OpenSAFELY Collaborative, Angel Y. S. Wong, Laurie A. Tomlinson, Jeremy P. Brown, William Elson, Alex J. Walker, Anna Schultze, Caroline E. Morton, David Evans, Peter Inglesby, Brian MacKenna, Krishnan Bhaskaran, Christopher T. Rentsch, Emma Powell, Elizabeth Williamson, Richard Croker, Seb Bacon, William Hulme, Chris Bates, Helen J. Curtis, Amir Mehrkar, Jonathan Cockburn, Helen I. McDonald, Rohini Mathur, Kevin Wing, Harriet Forbes, Rosalind M. Eggo, Stephen J. W. Evans, Liam Smeeth, Ben Goldacre, Ian J. Douglas

**Affiliations:** 1 Faculty of Epidemiology and Population Health, London School of Hygiene and Tropical Medicine, London, UK; 2 The DataLab, Nuffield Department of Primary Care Health Sciences, University of Oxford, Oxford, UK; 3 TPP, TPP House, Horsforth, Leeds UK; 4 NIHR Health Protection Research Unit (HPRU) in Immunisation, London, UK

**Keywords:** Warfarin, Direct oral anticoagulants, COVID-19

## Abstract

**Background:**

Thromboembolism has been reported as a consequence of severe COVID-19. Although warfarin is a commonly used anticoagulant, it acts by antagonising vitamin K, which is low in patients with severe COVID-19. To date, the clinical evidence on the impact of regular use of warfarin on COVID-19-related thromboembolism is lacking.

**Methods:**

On behalf of NHS England, we conducted a population-based cohort study investigating the association between warfarin and COVID-19 outcomes compared with direct oral anticoagulants (DOACs). We used the OpenSAFELY platform to analyse primary care data and pseudonymously linked SARS-CoV-2 antigen testing data, hospital admissions and death records from England. We used Cox regression to estimate hazard ratios (HRs) for COVID-19-related outcomes comparing warfarin with DOACs in people with non-valvular atrial fibrillation. We also conducted negative control outcome analyses (being tested for SARS-CoV-2 and non-COVID-19 death) to assess the potential impact of confounding.

**Results:**

A total of 92,339 warfarin users and 280,407 DOAC users were included. We observed a lower risk of all outcomes associated with warfarin versus DOACs [testing positive for SARS-CoV-2, HR 0.73 (95% CI 0.68–0.79); COVID-19-related hospital admission, HR 0.75 (95% CI 0.68–0.83); COVID-19-related deaths, HR 0.74 (95% CI 0.66–0.83)]. A lower risk of negative control outcomes associated with warfarin versus DOACs was also observed [being tested for SARS-CoV-2, HR 0.80 (95% CI 0.79–0.81); non-COVID-19 deaths, HR 0.79 (95% CI 0.76–0.83)].

**Conclusions:**

Overall, this study shows no evidence of harmful effects of warfarin on severe COVID-19 disease.

**Supplementary Information:**

The online version contains supplementary material available at 10.1186/s13045-021-01185-0.

## Background

People with severe COVID-19 disease have a high risk of thromboembolism [1, 2], and it is also known that lower levels of vitamin K could lead to pro-thrombotic conditions [3]. This might also lead to poorer outcomes among patients with COVID-19 treated with warfarin, which works by antagonising vitamin K. Unlike warfarin, the mechanism of action of direct oral anticoagulants (DOACs) is independent of vitamin K.

To date, there is limited evidence comparing outcomes from COVID-19 between patients treated with warfarin and those treated with DOACs. Current studies comparing the outcomes from COVID-19 between patients treated with warfarin and/or DOACs with non-anticoagulant users [4–7] limit the understanding of risks and benefits of prescribing different types of oral anticoagulants specifically in the context of the COVID-19 pandemic.

We therefore conducted a population-based cohort study to investigate the association between routinely prescribed warfarin and COVID-19-related outcomes, in comparison with those treated with DOACs. To minimise confounding by indication, we compared outcomes between patients treated for non-valvular atrial fibrillation (AF).

## Methods

### Study design

We conducted a population-based cohort study between 1 March 2020 and 28 September 2020.

### Data source

Primary care records managed by the software provider TPP were linked to SARS-CoV-2 antigen testing data from the Second Generation Surveillance System, COVID-19-related hospital admissions from the secondary uses service, and Office for National Statistics death data through OpenSAFELY, a data analytics platform created by our team on behalf of NHS England [8]. The data set analysed within OpenSAFELY is based on 24 million people currently registered with primary care practices using TPP SystmOne software, representing 40% of the English population. It includes pseudonymised data such as coded diagnoses, prescribed medications and physiological parameters.

### Study populations and exposure

**Fig. 1:**
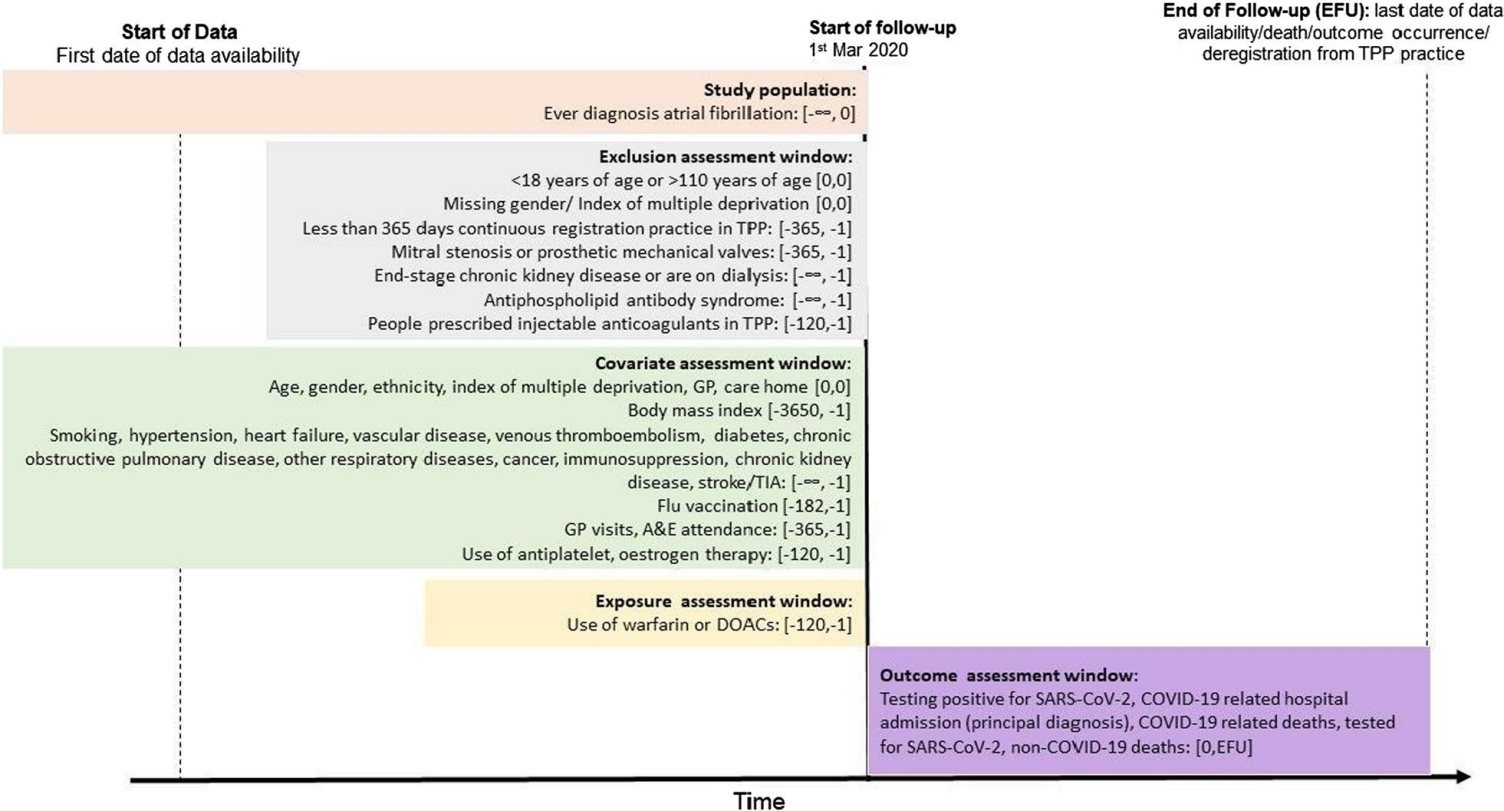
Study diagram

We first identified all patients with a diagnosis of AF on or before study start (1 March 2020) (Fig. 1). People with missing data for sex, Index of Multiple Deprivation, < 1 year of primary care records, or aged < 18 or > 110 and prescribed injectable anticoagulants 4 months before study start date were excluded. In addition, people with a record of mitral stenosis or prosthetic mechanical valves, chronic kidney disease stage V (estimated glomerular filtration rate < 15 mL/min or on dialysis), or antiphospholipid antibody syndrome before study start were also excluded in this study because DOACs are not recommended for use in these patient groups.

We defined participants as DOAC users if they were prescribed a DOAC as their latest OAC prescription in the 4 months before study start. The comparison group was people who were prescribed warfarin as the latest OAC prescription in the 4 months before study start date. If both warfarin and DOACs were prescribed on the same day as the latest prescription (*n* = 32), we classified them as warfarin users as a conservative estimate because warfarin is hypothesised to have a harmful effect on severe COVID-19 compared with DOACs.

### Outcomes and follow-up

The outcomes were (1) testing positive for SARS-CoV-2, (2) COVID-19-related hospital admission, and (3) COVID-19-related death (defined as the presence of ICD-10 codes U071 (confirmed COVID-19) and U072 (suspected COVID-19) anywhere on the death certificate). Testing outcomes were obtained from the UK’s Pillar 1 (NHS and Public Health England laboratories) and Pillar 2 (commercial partners) testing strategies and included results from polymerase chain reaction swab tests used to identify symptomatic individuals [9]. As pre-specified analyses, we also conducted negative control outcome analyses to examine the presence of residual confounding between warfarin and DOAC users. First, we anticipated that, within our population of people with non-valvular AF, there were unlikely to be marked differences in the likelihood of being tested for SARS-CoV-2 infection in relation to drug treatment with warfarin or DOAC. Therefore, we included being tested for SARS-CoV-2 as a negative control outcome to test our assumption. Second, we also included non-COVID-19 death, as differences in this outcome between DOAC and warfarin users could imply that differences in health characteristics had not been successfully controlled for. We conducted additional *post hoc* analyses to include cause-specific deaths as outcomes (i.e. death due to myocardial infarction, ischaemic stroke, venous thromboembolism, gastrointestinal bleeding and intracranial bleeding) to aid the interpretation of our results.

Follow-up for each cohort began on 1 March 2020 and ended at the latest of the outcome of interest in each analysis, deregistration from the TPP practice, death or study end date (28 September 2020) (Fig. 1).

### Covariates

Covariates were pre-specified, identified from a directed acyclic graph (DAG) approach (Additional file 1: Figure S1), including age, sex, obesity, smoking status, hypertension, heart failure, myocardial infarction, peripheral arterial disease, stroke/transient ischemic attack, venous thromboembolism, diabetes, flu vaccination, current antiplatelet use, current oestrogen and oestrogen-like therapy use, Index of Multiple Deprivation and care home residence. We identified covariates that are both associated with the exposure and the risk of severe COVID-19 outcomes either directly [8], or via venous thromboembolism [10, 11]. All codelists for identifying exposures, covariates and outcomes are openly shared at https://codelists.opensafely.org/ for inspection and reuse.

### Statistical methods

Baseline characteristics in each study were summarised using descriptive statistics, stratified by exposure status. We present adjusted cumulative incidence/mortality curves using the Royston–Parmar model (Additional file 1: Figure S2). We estimated hazard ratios (HRs) with 95% confidence intervals (CIs) using Cox regression with time since cohort entry as the underlying timescale. We accounted for competing risk by modelling the cause-specific hazard (i.e. censoring other deaths for COVID-19 death analysis and censoring any death for other outcomes analysis). We used graphical methods and tests based on Schoenfeld residuals to explore violations of the proportional hazards assumption.

We performed unadjusted models, models adjusted for age (using restricted cubic splines) and sex, and DAG-adjusted models (stratified by general practice).

### Quantitative bias analysis

We considered the possibility that if warfarin users had worse baseline health status they might act to lower their risk of SARS-CoV-2 infection through more risk-averse health behaviours (e.g. wearing face masks, avoiding close proximity to others) than DOAC users. Given that health behaviour is not captured in medical records, we conducted quantitative bias analyses to assess the sensitivity of our results to this potential unmeasured confounder.

We calculated the minimum strength of association required between an unmeasured confounder and one of exposure or outcome to move from the observed HR to a null bias-adjusted HR (aHR) (i.e. the E value) [12]. We also calculated the minimum strength of association required between unmeasured confounder and both of exposure and outcome to move from the observed HR to a null bias-aHR (i.e. the Cornfield condition) [12]. Furthermore, we calculated the minimum strength of association required to move from the observed protective associations to a bias-aHR of 1.2 because we hypothesised a harmful effect of warfarin in COVID-19-related outcomes.

### Sensitivity analyses

Table 1 shows the list of other sensitivity analyses.

**Table 1:** List of sensitivity analyses

Sensitivity analysis	Justification
1. In addition to the covariates identified by DAG, we included other covariates based on prior evidence of likely confounders such as chronic obstructive pulmonary disease, other respiratory diseases, cancer, immunosuppression, chronic kidney disease, general practice attendance rate in the year prior to cohort entry, and A&E attendance rate in the year prior to cohort entry in the fully adjusted models (stratified by general practice)	To test the robustness of the covariate selection
2. Additionally adjusted for ethnicity in DAG and fully adjusted models. In the fully adjusted models, additional covariates included chronic obstructive pulmonary disease, other respiratory diseases (not including asthma), cancer, immunosuppression, chronic kidney disease, General Practice attendance rate in the year prior to cohort entry, and Accident and Emergency attendance rate in the year prior to cohort entry	In the main analysis, we did not adjust for ethnicity as a sizable proportion of individuals with missing ethnicity (~23%). We undertook complete case analysis to address missing data
3. Repeated main analysis excluding people prescribed antiplatelets 4 months before study start date	To explore the impact of use of antiplatelet which can reduce the risk of blood clots
4. Repeated main analysis excluding people who were prescribed both warfarin and DOACs on the day of the latest OAC prescription	To assess the sensitivity of exposure definition
5. Repeated main analysis excluding people who ever had warfarin prescription 4 months before study start date in the DOAC group	As warfarin is hypothesised to have harmful effect on severe COVID-19 compared with DOAC, this analysis was to assess the sensitivity of exposure definition
6. Time-updated the OAC exposure variable	To evaluate the impact of national recommendation on drug switching from warfarin to DOACs due to COVID-19 pandemic [24]

Data management was performed using Python 3.8 and SQL, with analysis carried out using Stata 16.1. All study analyses were pre-planned unless otherwise stated. All code for data management and analyses in addition to the pre-specified protocol (https://github.com/opensafely/anticoagulants-research/blob/master/protocol/Protocol_%20Anticoag%20OpenSAFELY_v3.docx) are archived at: https://github.com/opensafely/anticoagulants-research.

### Role of the funding source

The funder of the study had no role in the study design, data collection, data analysis, data interpretation, or writing of the report. AJW, CEM, SB, WH, CB, JC, LS and BG had access to the raw data. The corresponding author had full access to all the data in the study and had final responsibility for the decision to submit for publication.

## Results

**Fig. 2:**
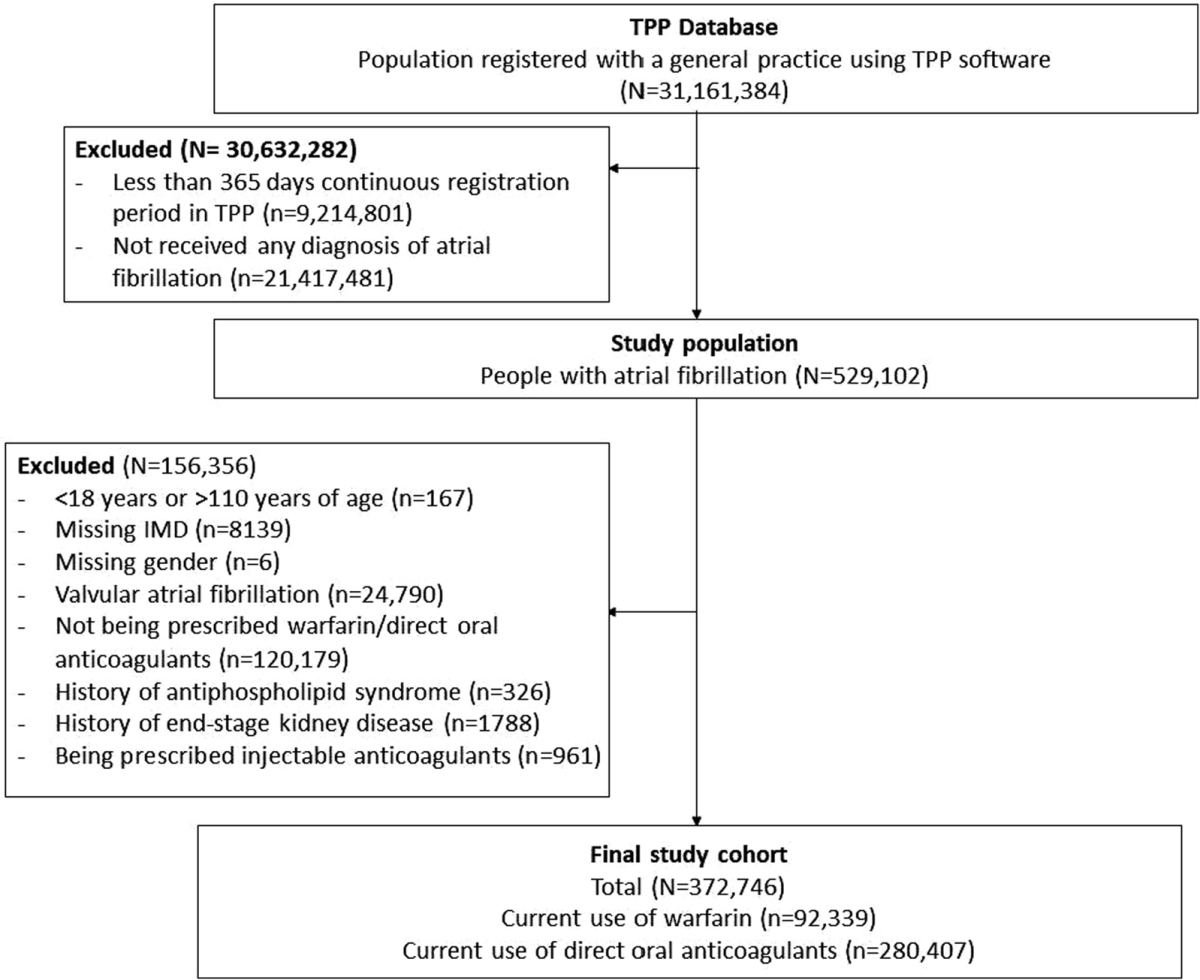
Flow chart of inclusion of participants

Figure 2 shows the flow chart of inclusion of people to develop the study cohort.

We included 92,339 warfarin users and 280,407 DOAC users (Table 2). Median age was 79 years (IQR, 73–85) among warfarin users and 78 years (IQR, 71–84) among DOAC users. A higher proportion of men were warfarin users (60.6%) than DOAC users (56.2%).

**Table 2:** Demographic and clinical characteristics

	Current use of direct oral anticoagulant	Current use of warfarin
Total	280,407	92,339
*Age as of 1 Mar 2020*		
18– < 40	510 (0.2)	112 (0.1)
40– < 50	2320 (0.8)	361 (0.4)
50– < 60	12,788 (4.6)	2245 (2.4)
60– < 70	42,407 (15.1)	9824 (10.6)
70– < 80	98,848 (35.3)	34,051 (36.9)
80+	123,534 (44.1)	45,746 (49.5)
Median, IQR	78 (71–84)	79 (73–85)
*Sex*		
Female	122,778 (43.8)	36,414 (39.4)
*Body mass index*		
< 18.5	5437 (1.9)	1199 (1.3)
18.5–24.9	72,658 (25.9)	21,998 (23.8)
25–29.9	94,621 (33.7)	31,981 (34.6)
30–34.9	57,590 (20.5)	19,592 (21.2)
35–39.9	24,032 (8.6)	8114 (8.8)
40+	12,586 (4.5)	4539 (4.9)
Missing	13,483 (4.8)	4916 (5.3)
*Ethnicity*		
White	201,046 (71.7)	66,800 (72.3)
Mixed	548 (0.2)	115 (0.1)
Asian/Asian British	3911 (1.4)	766 (0.8)
Black	1289 (0.5)	258 (0.3)
Other	1100 (0.4)	281 (0.3)
Missing	72,513 (25.9)	24,119 (26.1)
*Index of multiple deprivation*		
1 (least deprived)	57,570 (20.5)	17,703 (19.2)
2	56,881 (20.3)	18,400 (19.9)
3	55,654 (19.8)	19,056 (20.6)
4	54,758 (19.5)	18,615 (20.2)
5 (most deprived)	55,544 (19.8)	18,565 (20.1)
*Smoking status*		
Never	101,492 (36.2)	33,005 (35.7)
Former	161,752 (57.7)	54,463 (59.0)
Current	16,828 (6.0)	4834 (5.2)
Missing	335 (0.1)	37 (0.0)
Hazardous alcohol use	28,375 (10.1)	7819 (8.5)
Care home residence	8133 (2.9)	1039 (1.1)
*Comorbidities*		
Hypertension	195,078 (69.6)	66,888 (72.4)
Heart failure	71,427 (25.5)	26,926 (29.2)
Myocardial infarction	31,911 (11.4)	10,414 (11.3)
Peripheral arterial disease	14,273 (5.1)	5091 (5.5)
Stroke/transient ischaemic attack	60,271 (21.5)	18,470 (20.0)
Venous thromboembolism	19,927 (7.1)	8202 (8.9)
*Diabetes*		
Controlled (HbA1c < 58 mmols/mol)	61,178 (21.8)	23,893 (25.9)
Uncontrolled (HbA1c ≥ 58 mmols/mol)	22,672 (8.1)	7696 (8.3)
HbA1c not measured	838 (0.3)	298 (0.3)
COPD	36,189 (12.9)	11,272 (12.2)
Other respiratory diseases	16,444 (5.9)	4731 (5.1)
Cancer	49,488 (17.6)	16,240 (17.6)
Immunosuppression	1688 (0.6)	528 (0.6)
Chronic kidney disease	95,715 (34.1)	34,633 (37.5)
*Primary care consultations*		
Median, IQR	10 (6–17)	16 (9–27)
Min, Max	0, 432	0, 307
*A&E attendance*		
Median, IQR	0 (0–1)	0 (0–1)
Min, Max	0, 69	0, 45
Flu vaccination	220,153 (78.5)	78,558 (85.1)
*Medications*		
Oestrogen/oestrogen-like drugs	1652 (0.6)	361 (0.4)
Antiplatelets	19,030 (6.8)	4108 (4.4)

COPD, Chronic obstructive pulmonary disease

Current warfarin users were more likely to be obese, former smokers and have comorbidities than DOAC users, and had a greater number of primary care consultations and previous vaccinations. Current warfarin users were less likely to have a recent prescription for antiplatelets than DOAC users.

**Fig. 3:**
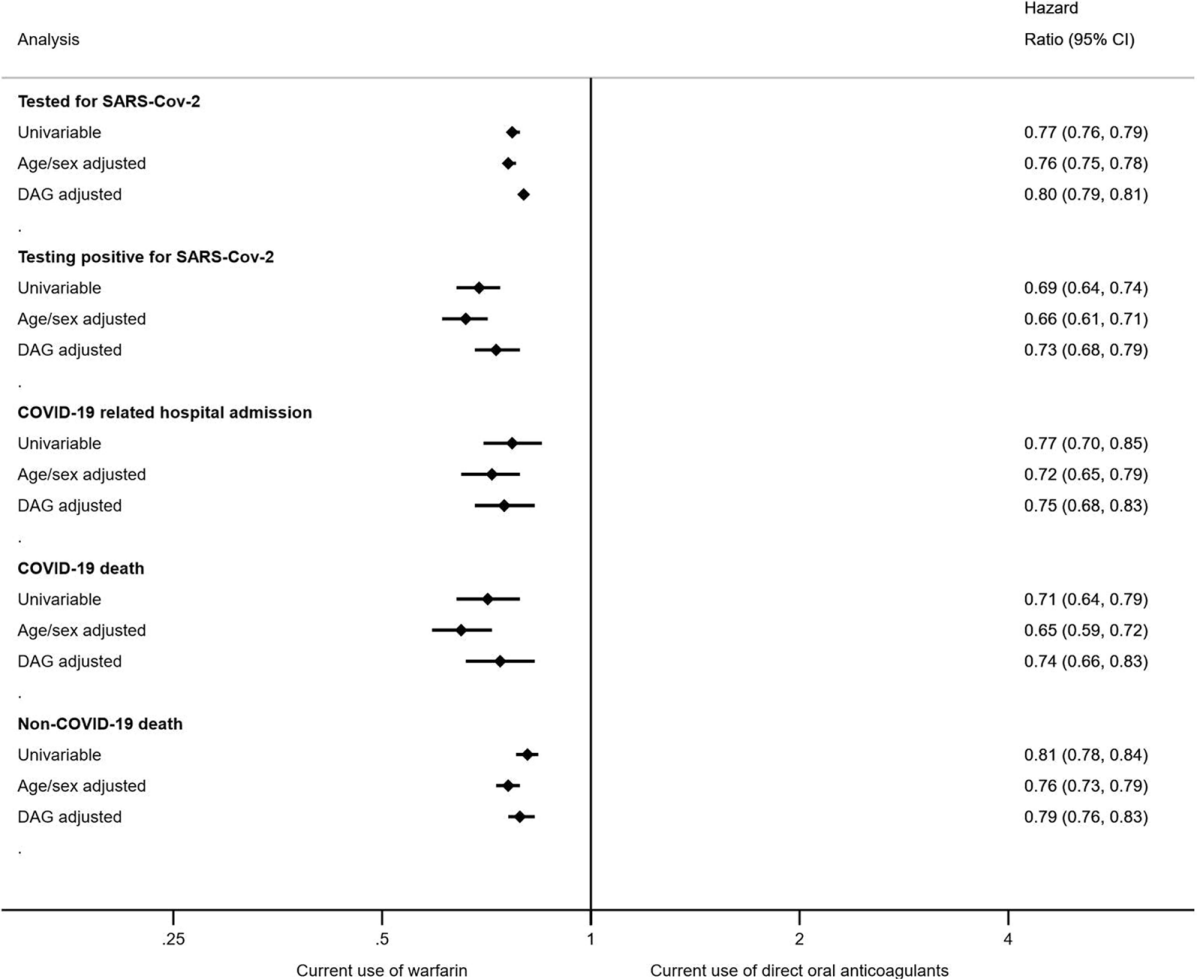
Hazard ratios of the association between current use of warfarin and COVID-19-related outcomes and non-COVID-19 deaths, versus direct oral anticoagulants in people with non-valvular atrial fibrillation

Figure S2 presents time to each outcome in adjusted cumulative incidence plots. We observed a lower risk for all outcomes associated with current use of warfarin versus current use of DOACs (Fig. 3 and Additional file 1: Table S1). A lower risk of testing positive for SARS-CoV-2 [unadjusted HR 0.69 (95% CI 0.64–0.74); DAG-aHR 0.73 (95% CI 0.68–0.79)], COVID-19-related hospital admission [unadjusted HR 0.77 (95% CI 0.70–0.85); DAG-aHR 0.75 (95% CI 0.68–0.83)], and COVID-19-related deaths [unadjusted HR 0.71 (95% CI 0.64–0.79); DAG-aHR 0.74 (95% CI 0.66–0.83)] were observed comparing current use of warfarin with current use of DOACs.

For the negative control outcome of being tested for SARS-CoV-2, the unadjusted HR for current use of warfarin was 0.77 (95% CI 0.76–0.79), with a DAG-aHR of 0.80 (95% CI 0.79–0.81) versus current use of DOACs. For non-COVID-19 deaths, the unadjusted HR was 0.81 (95% CI 0.78–0.84) with DAG-aHR of 0.79 (95% CI 0.76–0.83) comparing current use of warfarin with current use of DOACs.

In the *post hoc* analyses investigating cause-specific deaths, no differences in risk of each specific outcome were observed, although we had limited power for this analysis (Additional file 1: Table S1). The results of all other sensitivity analyses were broadly similar to those of the main analyses (Additional file 1: Tables S2–S6).

### Quantitative bias analysis

To potentially fully explain the observed non-null inverse associations, either DOAC use would need to be associated with at least 1.7–1.85 times increased risk of unmeasured risk-prone behaviour or risk-prone behaviour would need to be associated with 1.7–1.85 times increased risk of each COVID-19 outcome. Alternatively, both DOAC use and each COVID-19 outcome would need to be associated with the unmeasured risk-prone behaviour by associations ranging from 1.20 to 1.27 (Additional file 1: Table S7).

When we assumed an association between warfarin and each COVID-19 outcome of 1.2 (i.e. a harmful effect of warfarin compared with DOACs), either DOAC use would need to be associated with 2.25–2.41 times higher risk of unmeasured risk-prone behaviour, or unmeasured risk-prone behaviour would need to be associated with 2.25–2.41 times higher risk of each outcome to potentially fully explain the observed inverse associations. Alternatively, both DOAC use and each outcome would need to be associated with the unmeasured risk-prone behaviour by associations ranging from 1.45 to 1.52.

## Discussion

### Principal findings

In this large cohort study of people with non-valvular AF, we observed a lower risk of developing SARS-CoV-2 infection and hospital admission or death from COVID-19 among warfarin users compared to those prescribed DOACs. Surprisingly, we also observed that warfarin users were less likely to be tested for SARS-CoV-2.

The protective associations seen for warfarin versus DOACs in all patients with AF are surprising given the hypothesis of a possible harm with warfarin and generally more comorbidities among warfarin users than DOAC users. Also, our findings were non-specific, including an inverse association with being tested for SARS-CoV-2, and death from non-COVID causes. This might be partly explained by the behavioural differences between warfarin and DOAC users during COVID-19 pandemic. Although we cannot fully capture the behavioural differences between exposure groups in the database, we observed that warfarin users are less likely to be current smokers, had less hazardous alcohol use and more likely to have had flu vaccination than DOAC users, but the differences were small. Importantly, we adjusted for a range of confounders that had minimal impact to the estimates. We further performed quantitative bias analyses and found that an unmeasured risk-averse behaviour of moderate strength could potentially explain the observed inverse associations. Further studies that can account for behavioural differences between groups are required to confirm the findings as we cannot rule this out as a possible contributor to our findings.

Although we did not anticipate there was a difference in non-COVID-19 mortality between warfarin and DOACs, others have also observed a similar association using another UK primary care data set [13]. We therefore tried to explore non-COVID deaths further to understand whether this finding was driven by a possibly superior therapeutic effect of warfarin, but there were too few outcomes to draw conclusions. Whilst the aetiology of the protective association we observed between warfarin and COVID-19-related outcomes is unclear, importantly our results do not suggest that warfarin is associated with COVID-19-related harm compared with DOACs.

### Findings in context

Some early studies reported that elevated circulating D-dimer levels and prolonged prothrombin time were associated with mortality in people with COVID-19 disease [14, 15], suggesting COVID-19 coagulopathy [16]. The exact mechanism of hypercoagulability in COVID-19 disease is still not fully understood [17]. Therefore, anticoagulants have been proposed to be one of the investigational therapies in the management of COVID-19 disease, but the role of oral anticoagulants in severe COVID-19 outcomes remains unclear [18]. Further, effects of different types of oral anticoagulants in COVID-19 outcomes might be different due to the difference in mechanisms of action. Desphospho-uncarboxylated matrix Gla protein (dp-ucMGP) is an indirect marker of extrahepatic vitamin K status where high dp-ucMGP levels indicate low extrahepatic vitamin K status [19]. In a study of 135 hospitalised COVID-19 patients and 184 historical controls, it shows that dp-ucMGP was elevated in patients with COVID-19 compared with controls [3]. An even higher dp-ucMGP was observed in patients requiring intubation and mechanical ventilation or all-cause death. However, this study was of small sample size which required larger studies to confirm the association. In particular, the number of regular vitamin K antagonist users was very small (*n* = 15) to elucidate any role of vitamin K antagonists in severe COVID-19 outcomes. Another three cohort studies showed a trend of a lower risk of all-cause mortality associated with either prior use of warfarin or DOACs versus non-use of anticoagulants in patients with COVID-19 disease [4, 5, 7]. Another German cohort study reported no evidence of an association between DOACs and severe COVID-19 outcomes without including warfarin users into the analysis in people with non-valvular AF [6]. Notably, none of these studies directly compared the effects of warfarin with DOACs on COVID-19-related outcomes.

### Strengths and limitations

The greatest strength of this study was the power enabling us to examine the association between warfarin and various COVID-19-related outcomes versus DOACs as our data set included medical records from 24 million individuals. To our knowledge, this is the first population-based study comparing both the risk of COVID-related outcomes and cause-specific deaths between warfarin and DOACs. We also conducted quantitative bias analyses to explore the impact of unmeasured confounding to our observed results. The breadth of data available in primary care allows us to account for a wide range of potential confounders.

We recognise possible limitations. First, we could not eliminate residual confounding. Whilst differences in health behaviours and shielding between groups may partly explain our results, more studies are required to confirm these findings. Second, we are not able to capture any anticoagulant use during hospitalisation. Low molecular weight heparin or unfractionated heparin might be given for both warfarin and DOAC users with severe COVID-19 disease during hospitalisation to prevent venous thromboembolism. As the use of heparin during hospitalisation between warfarin and DOACs users is unlikely to be differential, this would merely lead to an underestimation of the effect without accounting for the anticoagulation use during hospitalisation in the analysis. Therefore, a lack of data on heparin during hospitalisation would not affect our interpretation that there is no evidence of harmful effects of warfarin on COVID-19 outcomes. Third, we do not know whether patients took the medications as prescribed. However, if there is a non-differential misclassification bias of exposure between warfarin and DOAC users, the estimates would be biased towards null. If DOAC users were more likely to adhere to their medication than warfarin users, the estimates would again tend to be biased towards null, with a hypothesis that warfarin has a harmful effect on COVID-19 outcomes. Moreover, we do not have data on inflammatory parameters to further explore the specific mechanism of severe COVID-19 disease due to coagulopathy in our cohort. It is recommended to incorporate inflammatory parameters into the outcome definition, so as to specifically investigate the impact of oral anticoagulants in severe COVID-19 diseases due to coagulopathy in future research. Lastly, we adjusted for the use of oestrogen and antiplatelets in our analysis that are both potentially associated with the oral anticoagulant type being prescribed and COVID-19 outcomes. However, we did not rule out the possibility of residual confounding due to other drug combinations with oral anticoagulants that were not adjusted for.

## Conclusions

We found no evidence of a higher risk of severe COVID-19 outcomes associated with warfarin versus DOACs, providing reassurance about the safety of warfarin use among patients with indications for anticoagulation in the context of the COVID-19 pandemic. We do not recommend changes to ongoing anticoagulant therapy based on these results.

## Supplementary Information

**Additional file 1**. Additional figures and tables for main analyses and sensitivity analyses.

## Data Availability

All data were linked, stored and analysed securely within the OpenSAFELY platform. Detailed pseudonymised patient data are potentially re-identifiable and therefore are not shared. We rapidly delivered the OpenSAFELY data analysis platform without prior funding to deliver timely analyses on urgent research questions in the context of the global COVID-19 health emergency. Now that the platform is established, we are developing a formal process for external users to request access in collaboration with NHS England. Details of this process should be published shortly on the OpenSAFELY website. All code for data management and analyses in addition to the pre-specified protocol (https://github.com/opensafely/anticoagulants-research/blob/master/protocol/Protocol_%20Anticoag%20OpenSAFELY_v3.docx) are archived at: https://github.com/opensafely/anticoagulants-research.

## Abbreviations

AF: Atrial fibrillation
CI: Confidence interval
DAG: Directed acyclic graph
dp-ucMGP: Desphospho-uncarboxylated matrix Gla protein
DOACs: Direct oral anticoagulants
HR: Hazard ratio

## Declarations

### Information Governance

NHS England is the data controller; TPP is the data processor; and the key researchers on OpenSAFELY are acting on behalf of NHS England. This implementation of OpenSAFELY is hosted within the TPP environment which is accredited to the ISO 27001 information security standard and is NHS IG Toolkit compliant [20, 21]; patient data have been pseudonymised for analysis and linkage using industry standard cryptographic hashing techniques; all pseudonymised data sets transmitted for linkage onto OpenSAFELY are encrypted; access to the platform is via a virtual private network (VPN) connection, restricted to a small group of researchers, their specific machine and IP address; the researchers hold contracts with NHS England and only access the platform to initiate database queries and statistical models; all database activity is logged; only aggregate statistical outputs leave the platform environment following best practice for anonymisation of results such as statistical disclosure control for low cell counts [22]. The OpenSAFELY research platform adheres to the data protection principles of the UK Data Protection Act 2018 and the EU General Data Protection Regulation (GDPR) 2016. In March 2020, the Secretary of State for Health and Social Care used powers under the UK Health Service (Control of Patient Information) Regulations 2002 (COPI) to require organisations to process confidential patient information for the purposes of protecting public health, providing healthcare services to the public and monitoring and managing the COVID-19 outbreak and incidents of exposure [23]. Taken together, these provide the legal bases to link patient data sets on the OpenSAFELY platform. GP practices, from which the primary care data are obtained, are required to share relevant health information to support the public health response to the pandemic and have been informed of the OpenSAFELY analytics platform.
