# Supplementary material for: Association between warfarin and COVID-19-related outcomes compared with direct oral anticoagulants: population-based cohort study
Source: J Hematol Oncol. 2021 Oct 19;14:172. doi: 10.1186/s13045-021-01185-0 (PMC8525065; doi:10.1186/s13045-021-01185-0)
Supplement: Supplementary file 1 — Additional file 1. Additional figures and tables for main analyses and sensitivity analyses. [file 13045_2021_1185_MOESM1_ESM.pdf]

Additional file 1: Association between warfarin and COVID-19 related outcomes compared with direct oral anticoagulants: cohort study

Contents

Figure S1. Directed Acyclic Graph investigating COVID-19 related outcomes and non-COVID-19 deaths comparing current use of warfarin and direct oral anticoagulants 2

Figure S2. Time to COVID-19 outcomes and non-COVID-19 death in adjusted cumulative incidence plots 3

Table S1. Results in the DAG/Fully adjusted models comparing warfarin use with direct oral anticoagulant use in people with non-valvular atrial fibrillation. 6

Table S2. Additionally adjusted for ethnicity in DAG models. 8

Table S3. Excluded people prescribed antiplatelets 4 months before study start date. 9

Table S4. Excluded people who were prescribed both warfarin and direct oral anticoagulants on the day as the latest oral anticoagulant prescription. 10

Table S5. Excluded people who ever had warfarin prescription 4 months before study start date in the direct oral anticoagulant group 11

Table S6. Time-updated oral anticoagulant exposure variable. 12

Table S7. Bias-analyses for DAG-adjusted hazard ratios. 13

Figure S1. Directed Acyclic Graph investigating COVID-19 related outcomes and non-COVID-19 deaths comparing current use of warfarin and direct oral anticoagulants

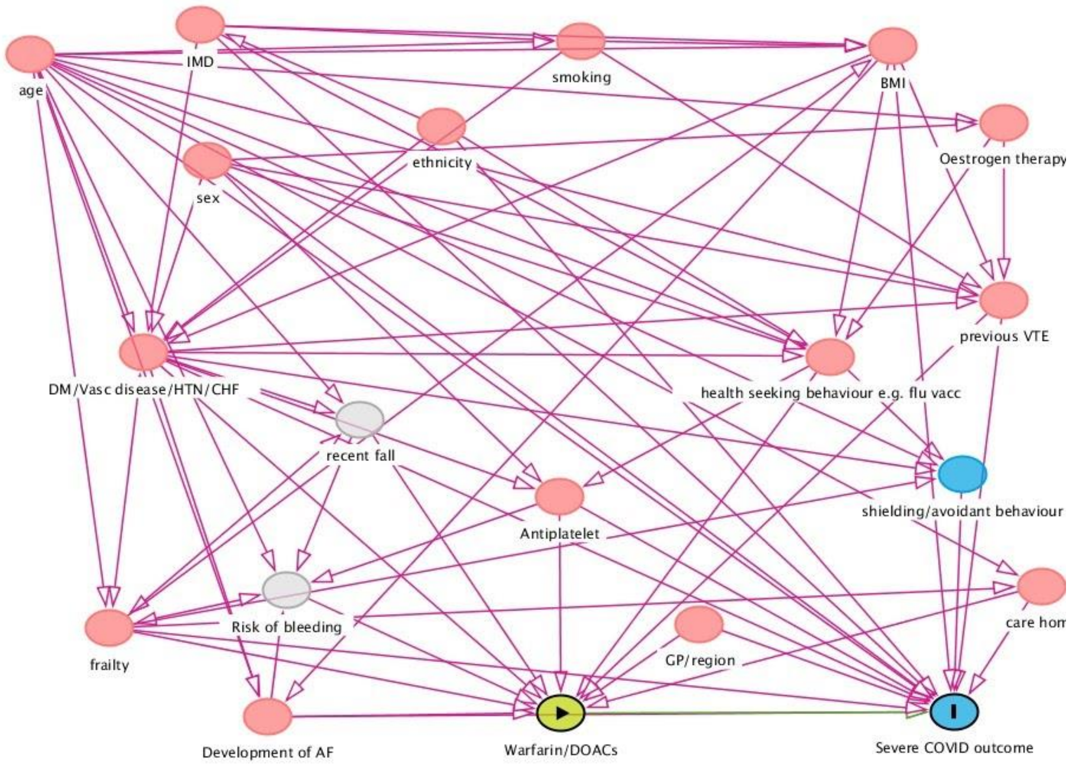

Figure S2. Time to COVID-19 outcomes and non-COVID-19 death in adjusted cumulative incidence plots.

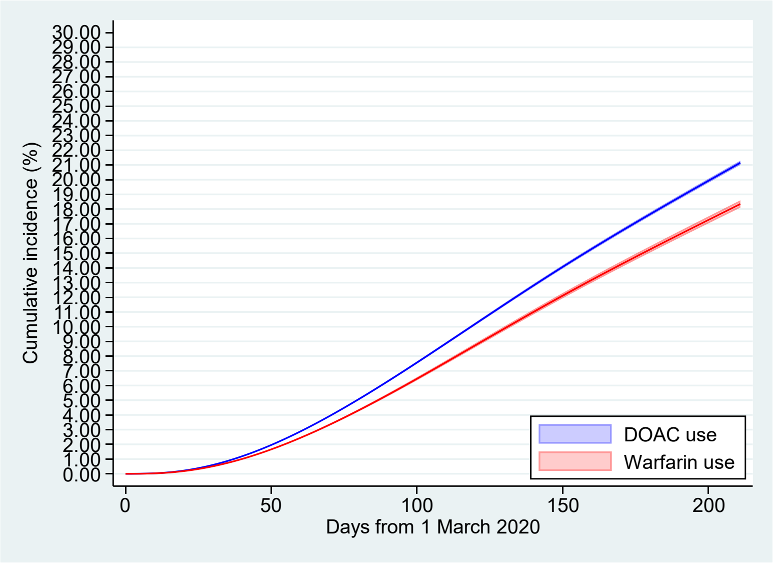

(a) Time to being tested for SARS-CoV-2

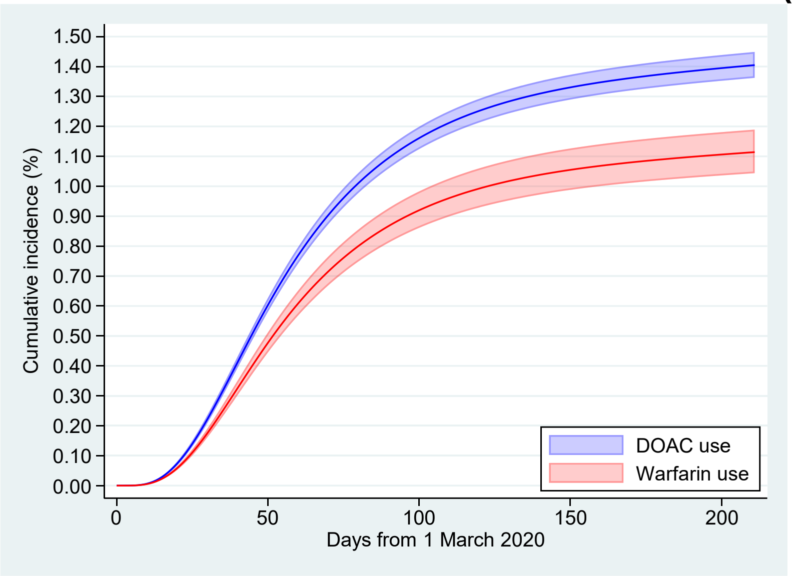

(b) Time to testing positive for SARS-CoV-2

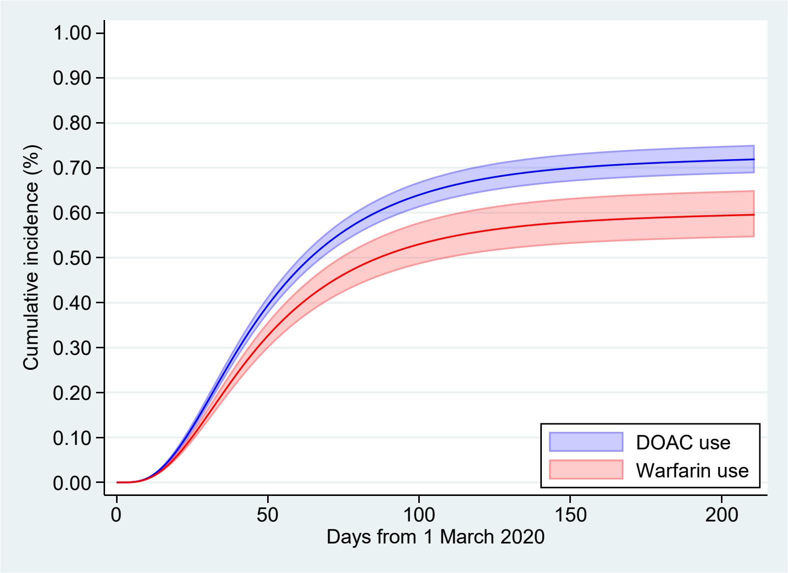

(c) Time to COVID-19 related hospital admission

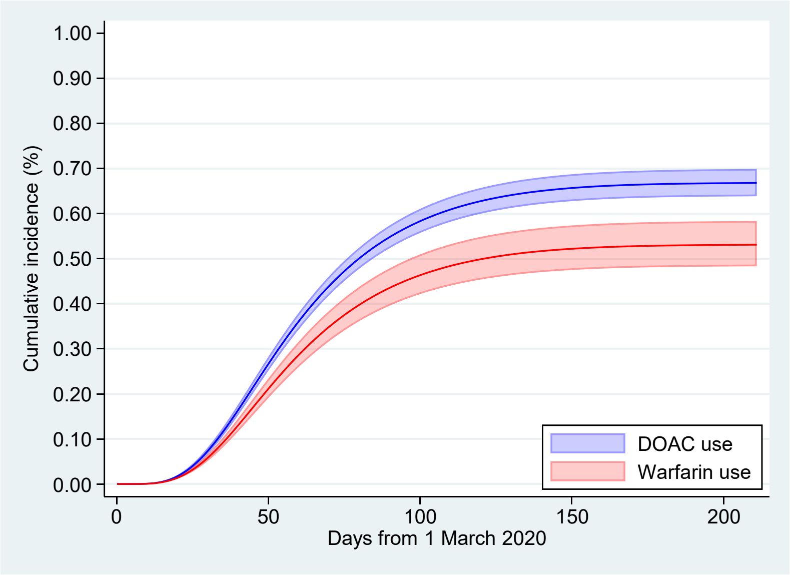

(d) Time to COVID-19 related deaths

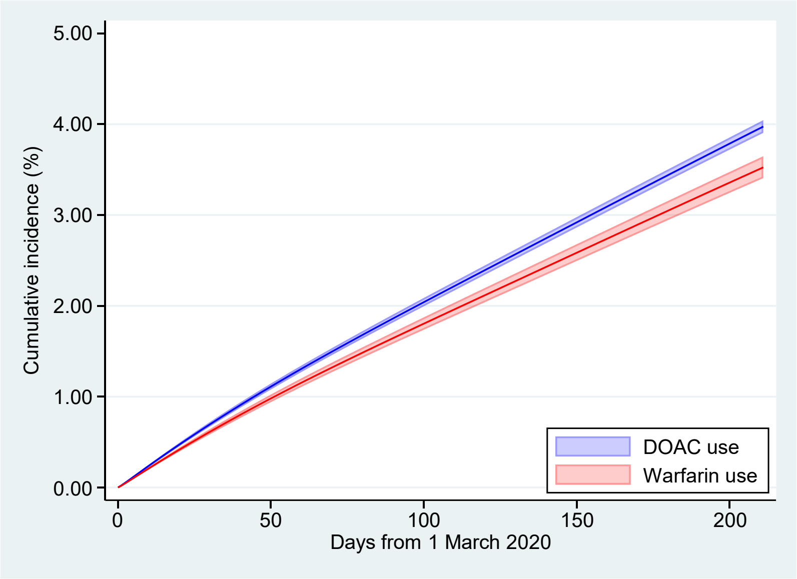

(e) Time to non-COVID-19 related deaths

These figures present cumulative incidence and mortality predicted from a Royston-Parmar model including all covariates from the fully-adjusted Cox model, with the baseline hazard parametrized as a 2-degrees-of-freedom cubic spline for COVID-19 related hospital admissions, testing positive for SARS-CoV-2, and COVID-19 related deaths, 3-degrees-of-freedom cubic spline for being tested for SARS-CoV-2, and non-COVID-19 deaths; predictions standardized to the covariate distribution of the exposure group.

Table S1. Results in the DAG/Fully adjusted models comparing warfarin use with direct oral anticoagulant use in people with non-valvular atrial fibrillation.

	Number of events	Total person-weeks	Rate per 1,000	Univariable		Age/Sex Adjusted		DAG Adjusted*		Fully adjusted[a]	
				HR	95% CI	HR	95% CI	HR	95% CI	HR	95% CI
**Tested for SARS-CoV-2**											
DOAC use	58123	7558624	7.69	1.00 (ref)		1.00 (ref)		1.00 (ref)		1.00 (ref)	
warfarin use	15354	2559790	6.00	0.77	0.76 - 0.79	0.76	0.75 - 0.78	0.80	0.79 - 0.81	0.85	0.84 - 0.87
**Testing positive for SARS-CoV-2**											
DOAC use	3882	8148585	0.48	1.00 (ref)		1.00 (ref)		1.00 (ref)		1.00 (ref)	
warfarin use	890	2709103	0.33	0.69	0.64 - 0.74	0.66	0.61 - 0.71	0.73	0.68 - 0.79	0.8	0.74 - 0.86
**COVID-19 related hospital admission**											
DOAC use	1973	8173823	0.24	1.00 (ref)		1.00 (ref)		1.00 (ref)		1.00 (ref)	
warfarin use	504	2714006	0.19	0.77	0.70 - 0.85	0.72	0.65 - 0.79	0.75	0.68 - 0.83	0.83	0.75 - 0.92
**COVID-19 death**											
DOAC use	1827	8195719	0.22	1.00 (ref)		1.00 (ref)		1.00 (ref)		1.00 (ref)	
warfarin use	431	2719891	0.16	0.71	0.64 - 0.79	0.65	0.59 - 0.72	0.74	0.66 - 0.83	0.81	0.73 - 0.91
**Non COVID-19 death**											
DOAC use	11188	8195719	1.37	1.00 (ref)		1.00 (ref)		1.00 (ref)		1.00 (ref)	
warfarin use	3007	2719891	1.11	0.81	0.78 - 0.84	0.76	0.73 - 0.79	0.79	0.76 - 0.83	0.88	0.84 - 0.92
**Myocardial infarction death[b]**											
DOAC use	413	8195719	0.05	1.00 (ref)		1.00 (ref)		1.00 (ref)		1.00 (ref)	
warfarin use	129	2719891	0.05	0.94	0.77 - 1.15	0.85	0.70 - 1.04	0.89	0.72 - 1.09	0.94	0.77 - 1.16
**Ischemic stroke death[c,d]**											
DOAC use	64	8195719	0.01	1.00 (ref)		1.00 (ref)		1.00 (ref)		1.00 (ref)	

warfarin use	11	2719891	<0.01	0.52	0.27 - 0.98	0.48	0.25 - 0.91	0.58	0.30 - 1.13	0.63	0.32 - 1.22
**Venous thromboembolism death[e]**											
DOAC use	21	8195719	<0.01	1.00 (ref)		1.00 (ref)		1.00 (ref)		1.00 (ref)	
warfarin use	6	2719891	<0.01	0.86	0.35 - 2.13	0.83	0.33 - 2.06	0.76	0.30 - 1.97	0.80	0.31 - 2.07
**Gastrointestinal bleed death[c]**											
DOAC use	50	8195719	0.01	1.00 (ref)		1.00 (ref)		1.00 (ref)		1.00 (ref)	
warfarin use	18	2719891	0.01	1.08	0.63 - 1.86	1.01	0.59 - 1.74	1.07	0.61 - 1.88	1.11	0.63 - 1.97
**Intracranial bleed death**											
DOAC use	191	8195719	0.02	1.00 (ref)		1.00 (ref)		1.00 (ref)		1.00 (ref)	
warfarin use	64	2719891	0.02	1.01	0.76 - 1.34	0.93	0.70 - 1.24	0.97	0.73 - 1.31	1.01	0.75 - 1.36

*Adjusted for age, sex, care home residence, obesity, smoking, hypertension, heart failure, myocardial infarction, peripheral arterial disease, stroke/transient ischemic attack, venous thromboembolism, diabetes, flu vaccination, antiplatelet use, oestrogen and oestrogen-like therapy use, and Index of Multiple Deprivation and stratified on general practice.

[a]Adjusted for age, sex, care home residence, obesity, smoking, hypertension, heart failure, myocardial infarction, peripheral arterial disease, stroke/transient ischemic attack, venous thromboembolism, diabetes, flu vaccination, antiplatelet use, oestrogen and oestrogen-like therapy use, Index of Multiple Deprivation, chronic obstructive pulmonary disease, other respiratory diseases, cancer, immunosuppression, chronic kidney disease, general practice attendance and A&E attendance and stratified on general practice.

[b]Due to low event count for parameters of oestrogen use, it did not converge in the model and was dropped from the main analysis.

[c]For outcomes of gastrointestinal bleed death and ischaemic stroke death, we classified people with a diabetes diagnosis but not having HbA1c measures in the past year as uncontrolled diabetes in DAG adjusted and fully adjusted models.as the parameter for not having HbA1c measures did not converge and people with a diabetes diagnosis but not having HbA1c measures in the past year, are likely to have uncontrolled diabetes due to their potential lack of monitoring and management of diabetes.

[d]Due to low event count for parameter of immunodeficiency, it did not converge in the model and was dropped from the main analysis.

[e]Due to low event count for parameter of antiplatelet use, and immunodeficiency, they did not converge in the model and were dropped from the main analysis.

Table S2. Additionally adjusted for ethnicity in DAG models.

	Number of events	Total person-weeks	Rate per 1,000	Univariable		Age/Sex Adjusted		DAG Adjusted with ethnicity*		DAG Adjusted without ethnicity[a]		Fully Adjusted with ethnicity[b]		Fully Adjusted without ethnicity[c]	
				HR	95% CI	HR	95% CI	HR	95% CI	HR	95% CI	HR	95% CI	HR	95% CI
**Tested for SARS-CoV-2**															
DOAC use	43365	5604356	7.74	1.00 (ref)		1.00 (ref)		1.00 (ref)		1.00 (ref)		1.00 (ref)		1.00 (ref)	
warfarin use	11367	1891645	6.01	0.77	0.76 - 0.79	0.76	0.75 - 0.78	0.8	0.78 - 0.82	0.8	0.78 - 0.82	0.85	0.84 - 0.87	0.85	0.84 - 0.87
**Testing positive for SARS-CoV-2**															
DOAC use	2965	6047824	0.49	1.00 (ref)		1.00 (ref)		1.00 (ref)		1.00 (ref)		1.00 (ref)		1.00 (ref)	
warfarin use	688	2003268	0.34	0.70	0.65 - 0.76	0.66	0.61 - 0.72	0.74	0.68 - 0.81	0.74	0.68 - 0.81	0.81	0.75 - 0.89	0.81	0.75 - 0.89
**COVID-19 related hospital admission**															
DOAC use	1530	6067451	0.25	1.00 (ref)		1.00 (ref)		1.00 (ref)		1.00 (ref)		1.00 (ref)		1.00 (ref)	
warfarin use	385	2007241	0.19	0.76	0.68 - 0.85	0.71	0.63 - 0.79	0.75	0.67 - 0.84	0.75	0.67 - 0.84	0.83	0.74 - 0.94	0.83	0.74 - 0.93
**COVID-19 death**															
DOAC use	1345	6084923	0.22	1.00 (ref)		1.00 (ref)		1.00 (ref)		1.00 (ref)		1.00 (ref)		1.00 (ref)	
warfarin use	318	2011890	0.16	0.72	0.63 - 0.81	0.65	0.57 - 0.73	0.73	0.64 - 0.83	0.73	0.64 - 0.83	0.81	0.71 - 0.92	0.81	0.71 - 0.92
**Non COVID-19 death**															
DOAC use	7800	6084923	1.28	1.00 (ref)		1.00 (ref)		1.00 (ref)		1.00 (ref)		1.00 (ref)		1.00 (ref)	
warfarin use	2109	2011890	1.05	0.82	0.78 - 0.86	0.76	0.72 - 0.80	0.8	0.76 - 0.84	0.8	0.76 - 0.84	0.89	0.85 - 0.93	0.89	0.85 - 0.93

*Adjusted for age, sex, ethnicity, care home residence, obesity, smoking, hypertension, heart failure, myocardial infarction, peripheral arterial disease, stroke/transient ischemic attack, venous thromboembolism, diabetes, flu vaccination, antiplatelet use, oestrogen and oestrogen-like therapy use, and Index of Multiple Deprivation and stratified on general practice.

[a]Adjusted for age, sex, care home residence, obesity, smoking, hypertension, heart failure, myocardial infarction, peripheral arterial disease, stroke/transient ischemic attack, venous thromboembolism, diabetes, flu vaccination, antiplatelet use, oestrogen and oestrogen-like therapy use, and Index of Multiple Deprivation and stratified on general practice

[b]Adjusted for age, sex, ethnicity, care home residence, obesity, smoking, hypertension, heart failure, myocardial infarction, peripheral arterial disease, stroke/transient ischemic attack, venous thromboembolism, diabetes, flu vaccination, antiplatelet use, oestrogen and oestrogen-like therapy use, Index of Multiple Deprivation, chronic obstructive pulmonary disease, other respiratory diseases, cancer, immunosuppression, chronic kidney disease, general practice attendance and A&E attendance and stratified on general practice.

[c]Adjusted for age, sex, care home residence, obesity, smoking, hypertension, heart failure, myocardial infarction, peripheral arterial disease, stroke/transient ischemic attack, venous thromboembolism, diabetes, flu vaccination, antiplatelet use, oestrogen and oestrogen-like therapy use, Index of Multiple Deprivation, chronic obstructive pulmonary disease, other respiratory diseases, cancer, immunosuppression, chronic kidney disease, general practice attendance and A&E attendance and stratified on general practice.

Table S3. Excluded people prescribed antiplatelets 4 months before study start date.

	Number of events	Total person-weeks	Rate per 1,000	Unadjusted		Age/Sex Adjusted		DAG Adjusted*		Fully adjusted[a]	
				HR	95% CI	HR	95% CI	HR	95% CI	HR	95% CI
**Tested for SARS-CoV-2**											
DOAC use	53304	7057738	7.55	1.00 (ref)		1.00 (ref)		1.00 (ref)		1.00 (ref)	
warfarin use	14451	2449231	5.9	0.78	0.76 - 0.79	0.77	0.75 - 0.78	0.8	0.78 - 0.81	0.85	0.83 - 0.87
**Testing positive for SARS-CoV-2**											
DOAC use	3598	7597766	0.47	1.00 (ref)		1.00 (ref)		1.00 (ref)		1.00 (ref)	
warfarin use	852	2589587	0.33	0.7	0.65 - 0.75	0.66	0.61 - 0.71	0.74	0.68 - 0.80	0.8	0.74 - 0.87
**COVID-19 related hospital admission**											
DOAC use	1793	7621589	0.24	1.00 (ref)		1.00 (ref)		1.00 (ref)		1.00 (ref)	
warfarin use	477	2594380	0.18	0.78	0.71 - 0.87	0.73	0.66 - 0.80	0.75	0.68 - 0.84	0.83	0.75 - 0.92
**COVID-19 death**											
DOAC use	1700	7641320	0.22	1.00 (ref)		1.00 (ref)		1.00 (ref)		1.00 (ref)	
warfarin use	410	2599887	0.16	0.71	0.64 - 0.79	0.65	0.58 - 0.72	0.73	0.66 - 0.82	0.8	0.72 - 0.90
**Non-COVID-19 death**											
DOAC use	10331	7641320	1.35	1.00 (ref)		1.00 (ref)		1.00 (ref)		1.00 (ref)	
warfarin use	2821	2599887	1.09	0.8	0.77 - 0.84	0.75	0.72 - 0.78	0.78	0.75 - 0.82	0.87	0.83 - 0.90

*Adjusted for age, sex, care home residence, obesity, smoking, hypertension, heart failure, myocardial infarction, peripheral arterial disease, stroke/transient ischemic attack, venous thromboembolism, diabetes, flu vaccination, oestrogen and oestrogen-like therapy use, and Index of Multiple Deprivation and stratified on general practice.

[a]Adjusted for age, sex, care home residence, obesity, smoking, hypertension, heart failure, myocardial infarction, peripheral arterial disease, stroke/transient ischemic attack, venous thromboembolism, diabetes, flu vaccination, oestrogen and oestrogen-like therapy use, Index of Multiple Deprivation, chronic obstructive pulmonary disease, other respiratory diseases, cancer, immunosuppression, chronic kidney disease, general practice attendance and A&E attendance and stratified on general practice.

Table S4. Excluded people who were prescribed both warfarin and direct oral anticoagulants on the day as the latest oral anticoagulant prescription.

	Number of events	Total person-weeks	Rate per 1,000	Unadjusted		Age/Sex Adjusted		DAG Adjusted*		Fully adjusted[a]	
				HR	95% CI	HR	95% CI	HR	95% CI	HR	95% CI
**Tested for SARS-CoV-2**											
DOAC use	58123	7558624	7.69	1.00 (ref)		1.00 (ref)		1.00 (ref)		1.00 (ref)	
warfarin use	15346	2558921	6.00	0.77	0.76 - 0.79	0.76	0.75 - 0.78	0.8	0.79 - 0.81	0.85	0.84 - 0.87
**Testing positive for SARS-CoV-2**											
DOAC use	3882	8148585	0.48	1.00 (ref)		1.00 (ref)		1.00 (ref)		1.00 (ref)	
warfarin use	889	2708170	0.33	0.69	0.64 - 0.74	0.66	0.61 - 0.71	0.73	0.68 - 0.79	0.8	0.74 - 0.86
**COVID-19 related hospital admission**											
DOAC use	1973	8173823	0.24	1.00 (ref)		1.00 (ref)		1.00 (ref)		1.00 (ref)	
warfarin use	504	2713072	0.19	0.77	0.70 - 0.85	0.72	0.65 - 0.79	0.75	0.68 - 0.83	0.83	0.75 - 0.92
**COVID-19 death**											
DOAC use	1827	8195719	0.22	1.00 (ref)		1.00 (ref)		1.00 (ref)		1.00 (ref)	
warfarin use	430	2718957	0.16	0.71	0.64 - 0.79	0.65	0.58 - 0.72	0.74	0.66 - 0.82	0.81	0.72 - 0.90
**Non-COVID-19 death**											
DOAC use	11188	8195719	1.37	1.00 (ref)		1.00 (ref)		1.00 (ref)		1.00 (ref)	
warfarin use	3005	2718957	1.11	0.81	0.78 - 0.84	0.76	0.73 - 0.79	0.79	0.76 - 0.83	0.88	0.84 - 0.92

*Adjusted for age, sex, care home residence, obesity, smoking, hypertension, heart failure, myocardial infarction, peripheral arterial disease, stroke/transient ischemic attack, venous thromboembolism, diabetes, flu vaccination, antiplatelet use, oestrogen and oestrogen-like therapy use, and Index of Multiple Deprivation and stratified on general practice.

[a]Adjusted for age, sex, care home residence, obesity, smoking, hypertension, heart failure, myocardial infarction, peripheral arterial disease, stroke/transient ischemic attack, venous thromboembolism, diabetes, flu vaccination, antiplatelet use, oestrogen and oestrogen-like therapy use, Index of Multiple Deprivation, chronic obstructive pulmonary disease, other respiratory diseases, cancer, immunosuppression, chronic kidney disease, general practice attendance and A&E attendance and stratified on general practice.

Table S5. Excluded people who ever had warfarin prescription 4 months before study start date in the direct oral anticoagulant group.

	Number of events	Total person-weeks	Rate per 1,000	Unadjusted		Age/Sex Adjusted		DAG Adjusted*		Fully adjusted[a]	
				HR	95% CI	HR	95% CI	HR	95% CI	HR	95% CI
**Tested for SARS-CoV-2**											
DOAC use	57285	7469075	7.67	1.00 (ref)		1.00 (ref)		1.00 (ref)		1.00 (ref)	
warfarin use	15354	2559790	6	0.78	0.76 - 0.79	0.77	0.75 - 0.78	0.8	0.79 - 0.82	0.86	0.84 - 0.87
**Testing positive for SARS-CoV-2**											
DOAC use	3804	8050487	0.47	1.00 (ref)		1.00 (ref)		1.00 (ref)		1.00 (ref)	
warfarin use	890	2709103	0.33	0.7	0.65 - 0.75	0.66	0.62 - 0.71	0.74	0.69 - 0.80	0.81	0.75 - 0.87
**COVID-19 related hospital admission**											
DOAC use	1935	8075203	0.24	1.00 (ref)		1.00 (ref)		1.00 (ref)		1.00 (ref)	
warfarin use	504	2714006	0.19	0.78	0.70 - 0.86	0.72	0.65 - 0.80	0.76	0.68 - 0.84	0.84	0.76 - 0.93
**COVID-19 death**											
DOAC use	1792	8096758	0.22	1.00 (ref)		1.00 (ref)		1.00 (ref)		1.00 (ref)	
warfarin use	431	2719891	0.16	0.72	0.65 - 0.80	0.65	0.59 - 0.73	0.74	0.67 - 0.83	0.81	0.73 - 0.91
**Non-COVID-19 death**											
DOAC use	10949	8096758	1.35	1.00 (ref)		1.00 (ref)		1.00 (ref)		1.00 (ref)	
warfarin use	3007	2719891	1.11	0.82	0.79 - 0.85	0.76	0.73 - 0.80	0.8	0.77 - 0.83	0.89	0.85 - 0.92

*Adjusted for age, sex, care home residence, obesity, smoking, hypertension, heart failure, myocardial infarction, peripheral arterial disease, stroke/transient ischemic attack, venous thromboembolism, diabetes, flu vaccination, antiplatelet use, oestrogen and oestrogen-like therapy use, and Index of Multiple Deprivation and stratified on general practice.

[a]Adjusted for age, sex, care home residence, obesity, smoking, hypertension, heart failure, myocardial infarction, peripheral arterial disease, stroke/transient ischemic attack, venous thromboembolism, diabetes, flu vaccination, antiplatelet use, oestrogen and oestrogen-like therapy use, Index of Multiple Deprivation, chronic obstructive pulmonary disease, other respiratory diseases, cancer, immunosuppression, chronic kidney disease, general practice attendance and A&E attendance and stratified on general practice.

Table S6. Time-updated oral anticoagulant exposure variable.

	Number of events	Total person-weeks	Rate per 1,000	Unadjusted		Age/Sex Adjusted		DAG Adjusted*		Fully adjusted[a]	
				HR	95% CI	HR	95% CI	HR	95% CI	HR	95% CI
**Tested for SARS-CoV-2**											
DOAC use	60952	7903763	7.71	1.00 (ref)		1.00 (ref)		1.00 (ref)		1.00 (ref)	
warfarin use	12525	2214651	5.66	0.76	0.74 - 0.77	0.75	0.73 - 0.76	0.78	0.76 - 0.80	0.83	0.82 - 0.85
**Testing positive for SARS-CoV-2**											
DOAC use	3994	8535512	0.47	1.00 (ref)		1.00 (ref)		1.00 (ref)		1.00 (ref)	
warfarin use	778	2322177	0.34	0.68	0.63 - 0.73	0.65	0.60 - 0.70	0.71	0.66 - 0.77	0.78	0.72 - 0.84
**COVID-19 related hospital admission**											
DOAC use	2025	8562354	0.24	1.00 (ref)		1.00 (ref)		1.00 (ref)		1.00 (ref)	
warfarin use	452	2325475	0.19	0.76	0.69 - 0.84	0.71	0.64 - 0.79	0.73	0.66 - 0.81	0.81	0.73 - 0.90
**COVID-19 death**											
DOAC use	1888	8586646	0.22	1.00 (ref)		1.00 (ref)		1.00 (ref)		1.00 (ref)	
warfarin use	370	2328963	0.16	0.69	0.61 - 0.77	0.63	0.57 - 0.71	0.7	0.62 - 0.79	0.77	0.69 - 0.87
**Non-COVID-19 death**											
DOAC use	11802	8586646	1.37	1.00 (ref)		1.00 (ref)		1.00 (ref)		1.00 (ref)	
warfarin use	2393	2328963	1.03	0.74	0.71 - 0.77	0.7	0.67 - 0.73	0.73	0.70 - 0.76	0.81	0.77 - 0.85

*Adjusted for age, sex, care home residence, obesity, smoking, hypertension, heart failure, myocardial infarction, peripheral arterial disease, stroke/transient ischemic attack, venous thromboembolism, diabetes, flu vaccination, antiplatelet use, oestrogen and oestrogen-like therapy use, and Index of Multiple Deprivation and stratified on general practice.

[a]Adjusted for age, sex, care home residence, obesity, smoking, hypertension, heart failure, myocardial infarction, peripheral arterial disease, stroke/transient ischemic attack, venous thromboembolism, diabetes, flu vaccination, antiplatelet use, oestrogen and oestrogen-like therapy use, Index of Multiple Deprivation, chronic obstructive pulmonary disease, other respiratory diseases, cancer, immunosuppression, chronic kidney disease, general practice attendance and A&E attendance and stratified on general practice.

Table S7. Bias-analyses for DAG-adjusted hazard ratios.

	Bias-adjusted hazard ratio	Observed hazard ratio			E-value			Cornfield condition		
		Point estimate	Lower CI bound	Upper CI bound	Point estimate	Lower CI bound	Upper CI bound	Point estimate	Lower CI bound	Upper CI bound
Tested for SARS-CoV-2	1	0.80	0.79	0.81	1.81	1.85	1.77	1.25	1.27	1.23
Testing positive for SARS-CoV-2	1	0.73	0.68	0.79	2.08	2.30	1.85	1.37	1.47	1.27
COVID-19 related hospital admission	1	0.75	0.68	0.83	2.00	2.30	1.70	1.33	1.47	1.20
COVID-19 death	1	0.74	0.66	0.83	2.04	2.40	1.70	1.35	1.52	1.20
Tested for SARS-CoV-2	1.2	0.80	0.79	0.81	2.37	2.41	2.33	1.50	1.52	1.48
Testing positive for SARS-CoV-2	1.2	0.73	0.68	0.79	2.67	2.93	2.41	1.64	1.76	1.52
COVID-19 related hospital admission	1.2	0.75	0.68	0.83	2.58	2.93	2.25	1.60	1.76	1.45
COVID-19 death	1.2	0.74	0.66	0.83	2.63	3.04	2.25	1.62	1.82	1.45

As a simplification, we consider higher-risk health behaviour to be a binary variable and assume no interaction between the unmeasured confounder and measured covariates on the outcome. To apply the bias analysis formulas to hazard ratios, we assume the outcome is rare.
